# Comparison of effects between SMR/delta-ratio and beta1/theta-ratio neurofeedback training for older adults with Mild Cognitive Impairment: a protocol for a randomized controlled trial

**DOI:** 10.1186/s13063-018-3170-x

**Published:** 2019-01-29

**Authors:** Fabienne Marlats, Leila Djabelkhir-Jemmi, Eric Azabou, Marouane Boubaya, Sjaak Pouwels, Anne-Sophie Rigaud

**Affiliations:** 1 Department of Clinical Gerontology, Broca Hospital, Assistance Publique-Hôpitaux de Paris (AP-HP), Research TEAM EA4468, Paris Descartes University, Paris, France; 2 Clinical Neurophysiology Laboratory, Department of Physiology, Raymond Poincaré Hospital, Assistance Publique-Hôpitaux de Paris (AP-HP), INSERM U1173, University of Versailles Saint Quentin en Yvelines, Garches, France; 3 Clinical Research Unit, Avicenne Hospital, Assistance Publique-Hôpitaux de Paris AP-HP, Bobigny, France; 4 Department of Surgery, Franciscus Gasthuis and Vlietland, Rotterdam/Schiedam, The Netherlands

**Keywords:** Neurofeedback, Mild Cognitive Impairment, Electroencephalography, Alzheimer, Memory, Attention

## Abstract

**Background:**

Older adults with Mild Cognitive Impairment (MCI) are at high risk of progressing to Alzheimer’s disease (AD). Slowing down the effect of dementia by enhancing brain plasticity represents one of the most prominent challenges. Neurofeedback (NF) has shown promising results in improving working memory but has never been evaluated in people with MCI. We aim to examine whether NF training can decrease cognitive disorders, targeting memory, attention functions and brain electrical activity in elderly patients with MCI.

**Methods:**

In this single-blind, randomized controlled trial (RCT) protocol, we will investigate the effects of two NF training protocols on cognitive performances and on brain electrical activity. Sixty MCI patients will be assigned either to an intervention program or to psycho-pedagogical care as a control condition. Participants in the intervention group will attend 30 sessions of sensorimotor/delta-ratio NF training or beta1/theta-ratio NF training. Neuropsychological assessment, questionnaires and electroencephalography (EEG) assessment parameters will be used as dependent variables in three periods: at baseline (T0), immediately after the last NF training session at 4 months (T1) and at 3-month follow-up (T2). The primary outcome will be the change in attention measured with the Trail Making Test B. Secondary outcome will be the changes in cognitive performance and in EEG activities.

**Discussion:**

If the results of our study show improvement in cognitive performances of older adults with MCI, this non-invasive, low-cost technique may deserve better consideration as a therapeutic intervention to delay cognitive decline and dementia. Consequently, research in NF will need to review and develop the rigor of its application in gerontology.

**Trial registration:**

ClinicalTrials.gov, ID: NCT03526692. Registered on 16 May 2018.

**Electronic supplementary material:**

The online version of this article (10.1186/s13063-018-3170-x) contains supplementary material, which is available to authorized users.

## Background

The concept of Mild Cognitive Impairment (MCI) is an intermediate clinical stage between normal cognitive functioning and mild dementia, indicating a deviation from normal aging [1]. Cognitive changes are noticed by the affected person and/or their family but do not jeopardize the activities of daily life. As used today, MCI is a syndrome rallying diverse pathological disorders affecting older adults [2]. Albert et al. [3] suggested a classification of MCI patients which includes MCI core-clinical, MCI low-likelihood, MCI intermediate-likelihood, and MCI high-likelihood groups, founded on the positivity of amyloid and neuronal injury biomarkers such as cerebrospinal fluid (CSF), beta-amyloid protein 42 (Aβ42) and CSF tau or neuronal injury with the presence of magnetic resonance imaging (MRI)-demonstrable hippocampal atrophy. MCI patients with markers are considered at high risk of developing Alzheimer’s disease (AD) [4]. They provide high motivation for the scientific community to develop pharmacological and non-pharmacological interventions, in order to stop or prevent further cognitive decline and dementia.

### Neuropathophysiology of MCI and AD

Since improvement in algorithms in electroencephalography (EEG) offers a promising approach to developing a database for assessing MCI and AD subjects, several researchers have underscored links between EEG signal and AD/MCI. With age, fast rhythms have been found to decrease [5, 6]. Most commonly in the healthy aging process, EEG changes are not consistently observable but might involve a slight decrease in frequency and amplitude in the alpha band (8–12 Hz), an increase in delta (1–4 Hz) and theta (4–8 Hz) activities unlike young healthy subjects [7, 8]. In AD, theta and delta rhythms are abnormally more dominant in temporal regions of the brain than in other cerebral regions and posterior alpha rhythms significantly decrease [9–13]. Other promising studies in pathophysiology revealed that patients with MCI showed dominant theta activity in the parietal and temporal areas [14–16]. Moretti et al. showed in numerous studies that the abnormal increase in the power ratio alpha3/alpha2 in temporo-parietal brain regions is correlated with hippocampal atrophy in MCI and AD patients [17–19]. Amnestic Mild Cognitive Impairment (A-MCI) subjects present decreased power of the posterior alpha band correlated with cognitive decline [12, 20–22]. Dubois et al. emphasized the decrease in the theta/alpha ratio explained by an increase in alpha oscillations over time in prefrontal areas in people who have Aβ42 deposition and people with prodromal AD. Then, other studies focused on the analysis of the beta band (13–30 Hz) changes at the MCI stage [15]. In recent years, progress in the understanding of MCI and AD pathophysiology has opened several therapeutic perspectives and facilitated the design of more adequate rehabilitation programs [23–27]. Interest in brain-training programs based on sustained cognitive exercises is rising [28, 29]. Non-pharmacological cognitive interventions have been recognized as an efficient method of enhancing cognitive and functional abilities of MCI [30–32].

### Neurofeedback

Neurofeedback (NF), also called electroencephalography Biofeedback (EEG Biofeedback) is one of the promising techniques that received international accreditation boards’ and treatment recommendations of evidence level A from the American Academy of Pediatrics for attention-deficit hyperactivity disorder (ADHD). Other promising results have been found for therapeutic efficacy in autism [33], patients with pharmaco-resistant epilepsy [34], and traumatic brain injury [35]. Neurofeedback uses real-time displays of brain activity—most commonly EEG—to teach self-regulation of brain function. This technique, defined as a closed-loop application, helps individuals to control or modify their cortical activity through learned self-regulation. Precisely, it is operant behavioral training that aims at improving the cognitive functions and that affects regulation by modulating brain electrical activity. The patient receives feedback of acoustic or/and visual signals (graphic or video games/animations/movies) when their rhythmic cortical electrical activity of specific cortical areas has exceeded an upper threshold [36]. The subject has to improve the quality of the signal by a continuing focus in an absolute relaxing state and not exceed a determined upper threshold in a frequency band. The aims are to obtain better alertness and eliminate symptoms of anxiety, consequently inducing better behavior.

Among the protocols of NF training, the sensorimotor brain rhythm (SMR, 12–15 Hz) is an oscillatory rhythm recorded over central scalp regions, generated in a reticular-thalamo-cortical network [37, 38]. SMR/delta NF training involves increasing the synaptic strengths and sensitivity within this network while maintaining low delta activity to avoid sleep (delta, 0.5–4 Hz) [39]. It was suggested that SMR NF training might facilitate thalamic inhibitory mechanisms and block motor activity that interferes with information processing [37]. In accordance with this assumption, few studies found memory and attention improvements after SMR NF training [36, 40–42].

The beta1/theta protocol consists of beta1 dominance (12–16 Hz band) and reduction of theta oscillations (4–7 Hz band) in the central frontal area. Beta1 is described as responsible for logical thinking, concentration, memory and emotional status [43]. Beta2 can be sign of anxiousness; NF training within this range might be conducted with prudence. Theta rhythm is associated with neurological and psychological functions in the limbic system, it serves regulatory control of arousal, affective and mental states [44]. Its excessive presence in frontal areas while the person is awake, indicates a decrease in vigilance with concentration and attention disorders. Thus, an increase in the central frontal beta1 band and a decrease in the central frontal theta band can be linked with an increase in vigilance and concentration and a decrease in diurnal sleepiness [43, 45].

Recently, several studies focused on investigating the benefits of NF training on cognitive functions in elderly subjects. It was shown that elderly patients, who completed a NF training program after stroke to generate SMR modulation on central regions of brain, improved in declarative learning memory [40, 46–50]. Other studies demonstrated increasing performance in working memory in healthy aging with a theta/alpha protocol training on Fz [29, 51] or with an upper alpha training in central regions of the brain [52]. Luijmes et al. [53] observed an increase in learning memory, recognition and recall of information after NF treatment in patients with AD. From a recent pilot study conducted by our research team at the Broca University Hospital in Paris, we investigated the effects of SMR/theta NF training on cognitive performance in elderly patients with MCI. Results showed that cognitive profits were minor, but anxiety decreased and quality of life and sleep improved. EEG data analyzed after the training program indicated that slow frequency bands decreased while alpha and sensorimotor rhythms were more dominant than in pre-training. Furthermore, this study enabled us to identify a few limits in the EEG NF application for older adults with MCI. These preliminary results allowed us to suggest some recommendations to avoid them such as the need to adapt the NF tasks, give explicit information about NF technique and all supplies, and to adjust the threshold to the cognitive and specific profile of the patient.

9pt?>Neurofeedback training seems to be a promising aproach in patients with AD or neurological disorders if this technique is adapted to elderly patients with MCI. On this basis, it would be relevant to further this technique in subjects at risk of developing AD. Based on the studies of Reichert et al. [47], Kober et al. [40] and Luijmes et al. [53], our objective is to examine: (1) the effects of SMR/delta NF training and the beta1/theta-ratio protocols on attention and memory performances in subjects with MCI, (2) to verify whether MCI patients exhibit a decrease or an increase in EEG activities in a resting state after SMR/delta NF training and/or alpha/theta-ratio NF training and (3) to assess the effects of the two training protocols on other cognitive and mood measures.

We hypothesize that (1) central frontal beta1/theta-ratio NF training might be effective in improving attention capacities whereas the SMR/delta protocol in Cz might be effective in improving memory performances and (2) changes in EEG patterns in post-SMR/delta-ratio and beta1/theta-ratio NF training would be observed in the intervention group but not in the control group. This paper has been conceived under consideration of the spirit guidelines (Additional file 1).

## Methods

### Study design

**Fig. 1:**
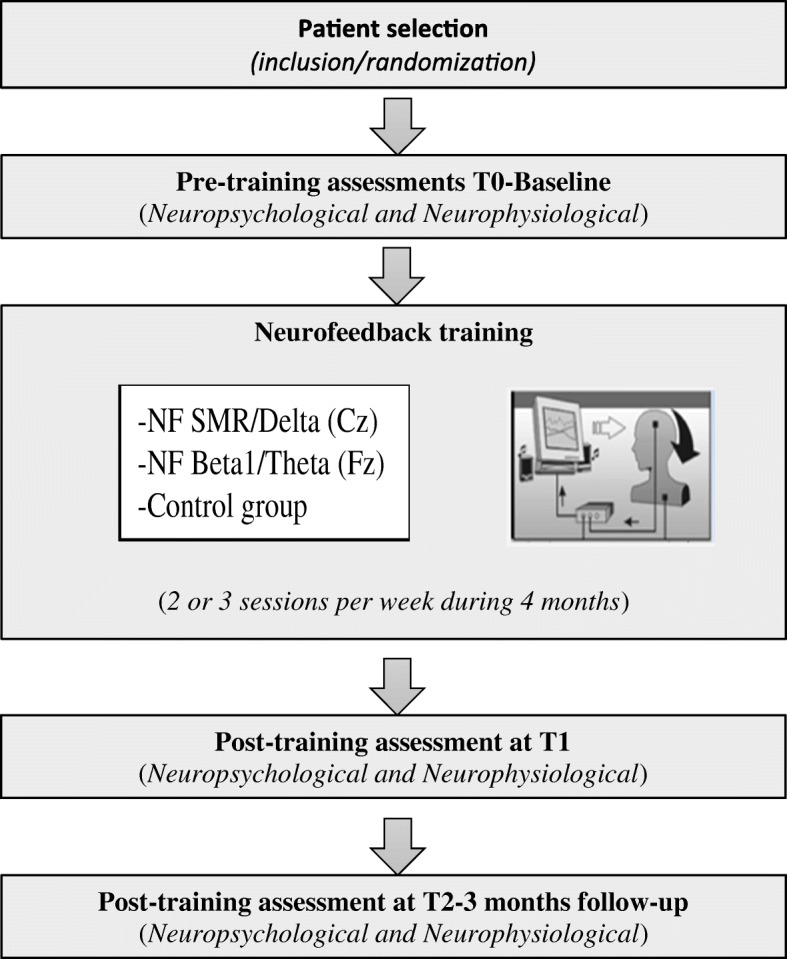
Experimental design, representation of the assessment and intervention

This study is a prospective, randomized controlled and single-blinded clinical trial as shown in Fig. 1. This study will be reported in accordance with both the Consolidated Standards of Reporting Trials (CONSORT) Statement, the CONSORT Statement for non-pharmacological treatment interventions and respecting Neurofeedback Protocol Guidelines [45]. One of the main investigators will inform patients, verbally and in a written document, of the objectives of the study, the project progress with EEG and neuropsychological assessments, and the potential risks. Each participant will be asked to sign a written consent form to ensure their voluntarily participation along with the information that they have right to discontinue the project any time without penalty and their agreement for the use of collected data. Signed consent forms will be kept by the main investigators.

### Randomization

After screening and obtaining their written consent, participants who are deemed eligible for the study will be enrolled and the randomization schedule will be designed to yield an assignment ratio of 1:1:1 between the three arms. Randomization will be centralized (CleanWEB; Telemedicine Technologies) and not stratified by blocks of variable size. No one from any group will know if they belong either to an experimental group or the control group (Fig. 1).

### Sample size

The sample size calculation was estimated on the basis of our feasibility study evaluating the effects of NF training on cognitive performances in MCI subjects (registered at ClinicalTrials.gov under the identifier: NCT03526692, on 16 May 2018). In an analysis of covariance study (ANCOVA), sample sizes of 20 are obtained for each of the three groups whose expected averages of TMT B are 140 in the control group and 105 s in two intervention groups with a standard deviation of 45. The supposed R-squared of the model is 0.20. The total sample of 60 subjects has a power of 80% to detect differences between means using an F test with a significance level of 0.05.

### Participants

Sixty older adults with MCI will be recruited by the geriatric medicine physicians through the outpatient memory service from Broca University Hospital in Paris. All participants will be eligible if they meet MCI criteria according to Petersen et al. [54]; are aged 60 years or over; have a Mini Mental State Examination (MMSE) [55] score > 24; a subjective memory complaint confirmed by an informant; perform at/or below 1.5 standard deviations from the mean for age and education-matched norms on more than one of the neuropsychological tests; preserve activity of daily living; and absence of dementia. The exclusion criteria will include patients with epilepsy, stroke, tumor, head trauma, personality disorders, and psychiatric disorders with the exception of mild depression using the GDS (Geriatric Depression Scale, normative score: normal 0–9, mildly depressed 10–19, very depressed 20–30) [56]. Participation in other trials will be prohibited during the study.

### Intervention

#### Neurofeedback training

Researchers have improved NF methodology, especially the determination of the criteria for the best use of this technique. We consider some essential technical aspects: the quality of the signal, the choice of the reward threshold, the type and number of positive reinforcements, the number of sessions, the duration of a session, task instructions and motivation given to the subject before and after the training session [45].

The sensorimotor rhythm is activated in central brain regions [57, 58]. Thus, EEG signals for SMR/delta-ratio training will be recorded from channel Cz according to the International 10–20 system and the instructions in the SMR NF protocol which suggest recording from C3, C4 or Cz. A-32 channel system (EEGDigiTrack Biofeedback Plus module, Inc. Elmiko Medical sp. z. o. o. (Ltd), Warsaw, Poland) will be used for SMR NF training. The delta rhythm is also recorded and, in this case, SMR is stimulated while delta waves are suppressed to avoid sleep.

For the second NF protocol beta1 (12–21 Hz)/theta (4–8 Hz), we aim to decrease this ratio by promoting beta1 and reducing theta. Training will be recorded in Fz, according to the international 10–20 system [45].

The ground electrode will be recorded at the right mastoid and the reference at the left mastoid. All impedances will be maintained below 5 KOhms (kΩ) and two inhibit bands will be used to avoid the augmentation of the signals by muscle artifacts such as, movements, coughs, jaw contraction, eye blinks (0.5–2 and 43–59 Hz). When the beta2 rhythm amplitude level increases, it usually means muscular artefact or an increase in the patient’s stress and tension levels, so the EEGDigiTrack System will suggest training and suppressing it.

**Fig. 2:**
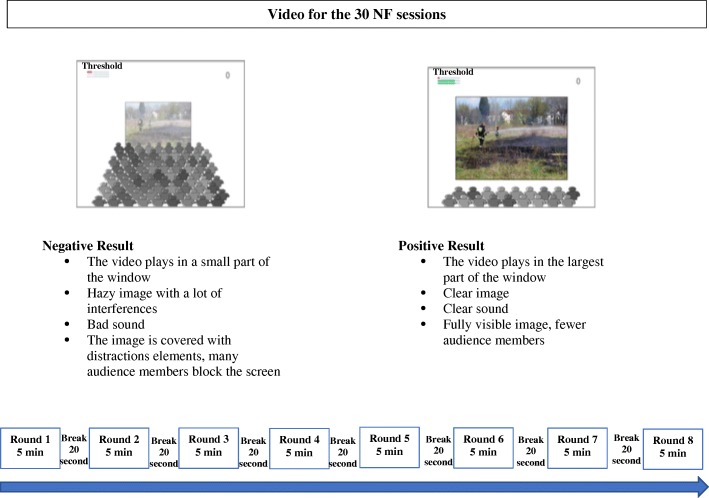
Description of a 40-min neurofeedback session with a historical reportage. Note reference: EEGDigiTrack, Inc. Elmiko Medical

The NF experiment will consist of 30 sessions, twice or three times a week during a maximum of 4 months. From our previous pilot study described above and carried out by our multidisciplinary team (data unpublished), we noticed that the nature of the task in NF training was very important to keep motivation and concentration in our sample. To avoid sleepiness or annoyance, we chose to keep the video of historical reportage as a NF task that had been unanimously appreciated. Thus, during the 30 sessions, videos of historical reportage will be watched for 40 min, divided in eight rounds of 5 min with 20-s break between. Session will last 45 min (Fig. 2). The breaks prevent drowsy episodes and permit the recommencing of sustained attention. The rest of the video will be shown at the next training. The subject receives visual and auditory feedback. To modulate brain activity in the SMR frequency band or in the beta1 band, the video will indicate a bad contrast and a particular sound each time the thresholds are not reached. The degree of difficulty will be manually adjusted by defining the thresholds. As a second feedback, a horizontal bar will be presented on the corner of the patient’s screen showing their real-time SMR power, delta power, beta1 power and theta power according to the threshold. When the expected band power reaches the predefined threshold, the color of the bar will change from red to green. Participants are instructed to try to voluntarily increase this bar to the green color. Neurofeedback training will be conducted by a PhD candidate experienced in neuropsychology, neurophysiology and NF.

A few conditions are recommended during training. For example, the room should be as quiet as possible and shaded; fluorescent lighting during the training must be avoided since it may cause interference; the patient should be at about 1.5 m from the monitor; the patient’s chair has to be stable with a headrest and should allow the patient to be in a comfortable position for muscle relaxing; only the technician and the patient are allowed to be in the room during the NF trainings.

#### Control group condition

Control group will receive a psycho-pedagogical program within the hospital, at the same frequency as experimental group. Each session will be organized using the same video material as for the NF training sessions. We will suggest that participants watch a historical report for 40 min with the recommendations to focus on dates, proper names and topics, then to answer verbally questions pertaining to the historical report for the last 20 min. As with the experimental group, the control group will undergo questionnaires, EEG recording and neuropsychological assessments at three time points: pre-training (T0), post training (T1) and follow-up (T2).

### Assessments

#### Cognitive outcomes

Neuropsychologists involved in data collection at any point of the assessment will be blinded to the condition assignment of each participant. They will complete cognitive assessments and questionnaires with a trained neuropsychologist blinded to the participants’ training condition, at baseline (T0), after the last NF training session at 4-month (T1) and at 3-month follow-up (T2), (see Fig. 2). Assessment duration will be done in one session for approximately 2 h in order to measure cognitive performances (see Table 1). Alternative test versions or forms will be used during post-NF training when possible to avoid learning effects due to repeated tests. Mood will be assessed by the Geriatric Depression Scale (GDS), a brief 30-item questionnaire to monitor depression over time in clinical settings [56]. Anxiety will be assessed with the Goldberg Anxiety Scale, the most commonly used rating scale to measure the severity of perceived anxiety symptoms [59].

**Table 1:** List of neuropsychological tests administered in pre and post-neurofeedback (NF) training

Neuropsychological test		
Global assessment of cognitive functions	Cognitive function	Description
• MoCa (2 versions) (range from 0 to 30)	Visuospatial/executive	Score between 21 to 25 = MCI
	Naming	
	Memory	
	Attention	
	Language	
	Abstraction	
	Orientation	
• MMSE (range from 0 to 30)	Brief quantitative cognitive measures	Score between 23 and 26/30 = MCI
	Orientation	
	Registration	
	Attention	
	Calculation	
	Recall	
	Language	
Episodic memory		
• RAVLT (4 lists of 15 words)	Declarative memory	A 15-noun word list, ability to encode, combine, store and recover verbal information
• Story Recall Test (A or B) recalls and a	Declarative memory	2 immediate delayed recall (30 min)
• DMS 48	Visual recognition memory	1st phase: implicit encoding 48 visual items and immediate recall; 2nd phase: 1 h delayed trial on choice recognition
• Rey-Osterrieth Complex Figure (or Taylor Complex Figure)	Visual declarative memory	30-min recall
Working memory and executive functions		
• TMTA (Trail Making Test part A)	Sustained attention	Capacity to maintain and attentional activity over a period of time
• TMTB (Trail Making Test part B)	Alternating attention	Capacity to shift in focus and tasks (B/A time)
• eCT	Executive function	Capacity to discriminate stimuli

Note: *MCI* mild cognitive impairment, *MoCa* Montreal Cognitive Assessment, *MMSE* Mini Mental State Examination, *RAVLT* Rey Auditory Verbal Learning Test, *DMS 48* Delayed Matching-to-Sample 48 items, *TMTA&B* Trail Making Test part A and part B, *eCT* Tablet-PC-based Cancellation Test. Rey, A. Story Recall [63]

#### Primary outcome

The primary outcome will be the change in attention measured by the Trail Making Test (TMT) part B between T0 and T2 compared to the control group. The TMT consists of measuring the time of the test performances [60, 61]. In TMT B, numbers and letters have to be alternately connected in the shortest time.

#### Secondary outcomes

The secondary outcome will include:

1. The changes in cognitive functions between T0 and T2 measured by:

- The score in part A of the Trail Making Test (TMT A), the number of errors in part A and B and the differences between B and A (B − A). In TMT A, 25 numbers have to be randomly distributed in sequential order as fast as possible
- The Rey Auditory Verbal Learning Test (RAVLT) assessing episodic memory. We will consider two performances: (1) the total learning score: the sum of all correct responses across five consecutive trials, (2) the delayed recall score: the number of correct responses at the last trial. The test consists in reading 15 words aloud to a participant who has to recall them freely. Four more consecutive learning trials are then performed in the same way. The word presentation order remains fixed across trials. After a 20-min delay period, the evaluator asks the participant to recall the words for the last time [62]
- The Story Recall Test assessing declarative memory. The test consists in reading a story with two immediate recalls and a delayed recall after 30 min [63, 64]
- The Delayed Matching-to-Sample 48-item (DMS 48) assesses visual recognition memory. The first phase is an implicit encoding of 48 visual items and an immediate recall. The second phase is a 1-h delayed trial on choice recognition [65]
- The Montreal Cognitive Assessment (MoCa), measures global cognitive efficiency whose visual/spatial, naming, memory, attention, language, abstraction and orientation [66, 67]
- The Mini Mental State Evaluation (MMSE) is a brief quantitative measure assessing orientation, registration, attention, calculation, recall and language [55]
- The Backward Digit Span of the Wechsler Adult Intelligent Scale-Fourth Edition (WAIS-IV) assesses working memory, processing speed/attention control [68]
- The Verbal Fluency (P letter, 2 min; animals, 2 min) measures verbal ability and executive control [69]
- The Rey-Osterrieth Complex Figure (ROCF) assesses planification/organization and visual memory and visual memory with a delayed recall (30 min) [70, 71]
- The Tablet-PC-based Cancellation Test (eCT) assesses executive function. It consists of simultaneously targeting stimuli while ignoring distractors as quickly as possible [55, 60, 67]
- Anxiety and depression will be assessed with the Goldberg Anxiety Scale and the Geriatric Depression Scale (GDS) [56, 59]

In order to reduce the risk that the test performance improvement may be due to learning effect of repeated assessments, we will use parallel versions of tests when available.

2) The changes in EEG activities in a resting state after SMR/delta NF training and/or alpha/theta-ratio NF training in treatment groups compared to the control subjects

#### EEG data acquisition

Electroencephalography will be recorded by a technician in EEG and EEG data will be analyzed by a neurophysiologist “blinded” to the participants’ training conditions. Neurofeedback training sessions will be performed by a doctoral student trained in EEG and EEG NF application.

For each participant, EEG will be recorded and an EEG power spectrum will be calculated in pre- (T0) and post-NF training/psycho-pedagogical care at T1 and T2. We will use an electrocap of 19 scalp locations (Electro-cap, International Inc. Elmiko Medical, Warsaw, Poland) according to the international 10–20 EEG placement system, in FP1, Fp2, F7, F3, Fz, F4, F8, T3, C3, Cz, C4, T4, T5, P3, Pz, P4, T6, O1 and O2 with reference at the left mastoid and the ground at the right mastoid. The recording will use the sampling frequency of 500 Hz and band-pass filter (0.01–100 Hz). Multichannel amplifier (EEGDigiTrack SimplEEG32, Inc. Elmiko Medical Sp. zo.o. Ltd., Warsaw, Poland) will be used. Impedance will be kept below 5 KΩ for EEG. EEG artifacts will be automatically rejected. Then, two baseline conditions will be recorded such as the eyes-open baseline (EOB) condition and an eyes-closed baseline (ECB) condition. Each condition will last for 5 min and will be repeated three times (30 min recording in total). During the EOB condition, participants will be required to minimize blinking to avoid eye-movement artifacts. During the ECB condition, participants will be asked to close their eyes but stay awake.

The power of the EEG rhythms, such as theta (4–7 Hz), alpha (8–12 Hz), SMR (12–15 Hz), low beta (13–20 Hz) and high beta (20–30 Hz) will be computed from the moving averaged EEG power spectra. The EEG data in pre-NF training and post-NF training will be analyzed offline using MATLAB R2011b.

#### Risks and side effects

EEG recording has no risks except skin irritation caused by cleaning the scalp with abrasive cleaning gel. EEG acquisition and NF equipment do not send an electrical current and only electrical output is recorded from the sensors. All equipment used in this research are certified as medical devices (class IIa. EU) for use with human subjects.

EEG NF has no medical risk and side effects are seldom observed but if they are they usually only include headache, fatigue and mild anxiety. They are quickly remediable and are explained by the difficulties of the NF training and concentration retention.

### Methods against bias

In order to guarantee blinded treatment allocation, an independent statistician engineer, not engaged in this investigation, will carry out randomization. Applying equal allocation ratios to all three conditions, the process will be stratified. Statistical analyses will be conducted using the conservative intent-to-treat population in order to avoid statistical bias. Primary and secondary outcomes, and EEG recordings will be assessed by trained investigators and technicians who will be blind to the patient’s study status.

Effect sizes and confidence intervals will be calculated as Standardized Mean Change Index for within-group comparisons and as Standardized Mean Index difference for between-group comparisons and corrected for small sample sizes.

### Data management

Technical specifications of the trial database, such as variable names, age, disease, frequency of visits and sessions will be predefined and documented in the database manual and in the electronic Case Report Form. All information on the Case Report Forms will be confidential and traceable to source documents in the patient’s file; the original documents will be kept by the main investigators of the study at the Broca University Hospital in Paris. At the end of the study, personal information will be deleted. The final trial dataset and disclosure of contractual agreements will be kept at the Broca University Hospital in Paris. Interim analyses on the acquired data will be conducted and presented in meetings and conferences. Results of the final analyses will be presented at scientific conferences and published in scientific journals.

### Statistical analysis

Analysis will be performed based on an intent-to-treat (ITT) principle; ITT analysis includes every subject who is randomized according to randomized treatment assignment. Baseline characteristics of the treatment groups will be summarized descriptively without any statistical tests of significance. Categorical variables will be described as numbers and percentages and continuous variables as means with standard deviation.

Neuropsychological performances, whose primary outcome and questionnaires scores will be compared between the SMR/delta-ratio NF group, the beta1/theta-ratio NF group and the control group by using the ANCOVA model. For analysis of primary outcome, the ANCOVA model includes the intervention group, the TMT B score and the number of errors. Pairwise comparisons will be performed with Bonferroni’s correction if group effect is significant. Statistical analysis will be applied to all outcome measures in a comparative analysis of data collected at baseline (T0), after the last NF training session at the 4-month (T1) and 3-month follow-ups (T2). A *T* test for paired data will be used for intra-group comparison. As few participants as possible should leave the program but patients who drop out should be followed up in order to record data as required by the intention-to-treat principle. Missing data will be imputed using multiple imputations by chained equations.

No interim analysis and no subgroup analysis were planned. All tests will be two-sided at a 0.2 significance level. All statistical analyses will be carried out using R statistical software version 3.3.2 (R Foundation for Statistical Computing, Vienna, Austria).

### EEG analysis

EEG data will be processed with MATLAB R2011b, MathWorks Inc., Natick, MA, USA.

We will calculate the power spectral density of EEG signals to extract the relative power (RP) and absolute power (AP) for each frequency band. By definition, the RP is a value that compares the AP within a frequency band with the total AP. So, relative theta, relative alpha, relative SMR and relative beta will be calculated in the central, frontal and temporal areas during the rest ECB and rest EOB conditions in pre-NF and post-NF training. The ANCOVA model will provide the differences in RP between these two times. When the difference is positive, it means that the RP in post-NF training for that channel is larger than the RP in pre-NF training. The variables will be used to assess differences between the SMR NF group and the control group.

The time schedule and analysis plan are illustrated in Table 2.

**Table 2:** Analysis plan and time schedule

Activity/month	Responsible	1st year				2nd year												3rd year				
		1	5	9	11	13	14	15	16	17	18	19	20	21	22	23	24	25	26	27	28	29
Project development	Main investigators	x	x	x	x																	
Ethical Committee approval	Main investigators		x																			
Protocol preparation	Main investigators	x	x	x	x																	
Recruitment	Main investigators					x	x	x	x	x	x											
Informed consent	Main investigators					x	x	x	x	x	x											
Participants selection	Main investigators					x	x	x	x	x	x											
Information meeting with patient	Main investigators					x	x	x	x	x	x											
Randomization	Engineer in biostatistics										x											
Neuropsychological assessments	Neuropsychologists										x				x			x				
Questionnaire assessments	Neuropsychologists										x				x			x				
Electroencephalography recording	Technician specialized in EEG										x				x			x				
Neurofeedback training intervention	Main investigators										x	x	x	x	x							
Data collection	Main investigators					x	x	x	x	x	x				x							
Data analysis	Engineers in biostatistics										x				x			x	x			
Hypothesis testing	Main investigators + engineers in biostatistics														x			x				
Results and conclusion	Main investigators														x	x	x	x				
Dissemination of results (publication, conference, presentations)	Main investigators										x				x	x	x	x	x			

*EEG* electroencephalography

### Quality assurance

To ensure that a large number of participants finish the study and participate in as many sessions as possible with motivation, the PhD candidate specialized in electrophysiology, neuropsychology and gerontology will meet each participant to explain specific program information, show the equipment (EEG, and EEG NF), and give a demonstration of EEG NF training before assessment at baseline. All participants will receive times and contact details of study investigators if they have questions or encounter difficulties during the intervention program.

An independent technician specialized in EEG will record EEG in each patient. A neuropsychologist with full knowledge of all measurement scales and administration tests will ensure assessments and data collection. Neurofeedback training sessions will be ensured by a PhD candidate specialized in electrophysiology, neuropsychology and gerontology. In order to guarantee blinded treatment allocation, an independent engineer in biostatistics, not engaged in this investigation, will carry out statistical analysis and EEG analysis.

### Resources

The Broca University Hospital in Paris will provide access to neuropsychological tests, questionnaires, materials, computers and infrastructure that are necessary for conducting the study. Neurofeedback software and license EEGDigiTrack Biofeedback plus module and the multichannel amplifier EEGDigiTrack SimplEEG32 are provided by the company Inc. Elmiko Medical, Warsaw, Poland.

## Discussion

To our knowledge, this study is the first RCT using an SMR/delta-ratio NF group, a beta1/theta-ratio NF group of elderly patients with MCI. Design of our study is not the “gold standard” that would be a placebo-controlled, randomized, double-blind study. Nevertheless, research in NF has to consider the specificity of the application and the protocol to use the technique correctly. Even if different methods are used in different laboratories, the technician trained in NF needs to have control of the protocol and the modulation of the threshold during each training session. Accordingly, in our study, blinding technicians during NF training is not possible. In contrast, psychologists, neurophysiologist assessors, and the statistician analyst are blinded to the participants’ training conditions.

Patients can follow the training with instructions to understand the rules and goals of the activities. They cannot be totally blinded to the treatment condition.

In studies using sham NF training for the control group, the feedback features were signals from other subjects or from a mixture of noise. If we consider that NF training influences behavior, cognitive performance, alertness, mood and brain activity, then training from wrong NF signals might influence one or a few of these parameters in a positive or negative way. In any case, wrong NF signals induce changes, such as frustration of failure, while the subject’s concentration was positive. Incoherence of NF signals with the person’s behavior and alertness is an ethical scientific dimension that needs to be considered for future studies in NF.

We have not defined stopping criteria of the study because we do not expect side effects. EEG NF is a safe and well-established technique. However, the principal investigator will decide if the participants can continue or stop the study if side effects are reported.

The results of this study may be relevant in several aspects. First, there is a need for more research in the field of NF to develop rigorous application while recognizing the necessary adaptation to the conditions of elderly patients with MCI. Then, positive results in our pilot study will allow for the development of a larger population study and for experiments in other diseases, such as AD and epilepsy, in aging. This study will also enable us to obtain complete information regarding the correlation between neurophysiology and cognitive functions in elderly patients with MCI.

### Trial status

Recruitment will start in January 2019. The end of the trial is planned for February 2020.

## Additional file

Additional file 1: Standard Protocol Items: Recommendations for Interventional Trials (SPIRIT) 2013 Checklist: recommended items to address in a clinical trial protocol and related documents*. (DOC 124 kb)

## Abbreviations

AD: Alzheimer’s disease
ADHD: Attention-deficit hyperactivity disorder
ANCOVA: Analysis of covariance
CONSORT: Consolidated Standards of Reporting Trials
DMS 48: Delayed Matching to Sample 48 items
eCT: Tablet PC-based Cancellation Test
EEG: Electroencephalography
GDS: Geriatric Depression Scale
MCI: Mild Cognitive Impairment
MMSE: Mini Mental State Evaluation
MoCa: Montreal Cognitive Assessment
NF: Neurofeedback
RAVLT: Rey Auditory Verbal Learning Test
RCT: Randomized controlled trial
ROCF: Rey Osterrieth Complex Figure
SMR: Sensorimotor brain rhythm
WAIS IV: Weschler Adult Intelligent Scale-Fourth Edition
